# Creation and Validation of an Automated Registry for Outpatient Parenteral Antibiotics

**DOI:** 10.1093/ofid/ofae004

**Published:** 2024-01-12

**Authors:** Joseph Canterino, Maricar Malinis, Jing Liu, Nitu Kashyap, Cynthia Brandt, Amy Justice

**Affiliations:** Yale University School of Medicine, New Haven, Connecticut, USA; Yale University School of Medicine, New Haven, Connecticut, USA; Yale–New Haven Health, New Haven, Connecticut, USA; Yale University School of Medicine, New Haven, Connecticut, USA; Yale–New Haven Health, New Haven, Connecticut, USA; Veterans Affairs Heathcare System, West Haven CT; Yale University School of Medicine, New Haven, Connecticut, USA; Veterans Affairs Heathcare System, West Haven CT; Yale University School of Medicine, New Haven, Connecticut, USA

**Keywords:** informatics, OPAT, registry, value, Dashboard

## Abstract

Existing outpatient parenteral antibiotic therapy (OPAT) registries are resource intensive, and OPAT programs struggle to produce objective data to show the value of their work. We describe the building and validation of an automated OPAT registry within our electronic medical record and provide objective data on the value of the program. Variables and outcomes include age, sex, race, ethnicity, primary insurance payor, antibiotic names, infection syndromes treated, discharge disposition, 30-day all-cause readmission and death rates, complications, and an estimate of the hospital days saved. Records for 146 OPAT episodes were reviewed manually to validate the registry. Data were displayed in a dashboard within the electronic medical record. Over the 4-year time frame, our registry collected 3956 unique patients who completed 4710 episodes (approximately 1200 episodes per year). A total of 400 complications during OPAT were identified. All variables had an accuracy of >90% on validation. The OPAT program resulted in a reduction in hospital length of stay by 88 820 days, or roughly 22 000 days per year. We intend our registry to serve as a blueprint for similar OPAT programs with limited administrative resources. Wider application of our system would allow for easier aggregation and comparisons of OPAT practice and address the lack interinstitutional standardization of OPAT data and outcomes.

## Teaser

We were able to successfully build and validate an automated (data collected without any manual curation) outpatient parenteral antimicrobial therapy (OPAT) registry that displays outcome metric changes over time and demonstrates the value of the OPAT team.

OPAT is the administration of intravenous antibiotics in the ambulatory setting, including in the home, at a skilled nursing facility, or at an infusion center. OPAT has many advantages over remaining in the hospital for treatment, including higher patient satisfaction [1, 2], increased availability of hospital beds [2] and cost savings [1]. Owing to their high complexity and treatment monitoring requirements, patients receiving OPAT are ideally managed by a team or program dedicated to overseeing their care in the community. These programs typically consist of infectious diseases (ID) physicians, pharmacists, nurses, and advanced practice providers. However, only a minority of ID physicians report having access to an OPAT program, data analytics or administrative support [3]. In addition, no universal quality metrics have been established for OPAT programs in the United States to judge their practice.

Despite these obstacles, some institutions have managed to create their own OPAT registries to collect patient demographic information, infections being treated, antibiotics used, adverse events, and clinical and programmatic outcomes [1, 2, 4–8]. While these registries have provided useful snapshots of OPAT practice and performance, they are resource intensive (often generated from the manual curation of information), and without standard methods for data collection it is difficult to draw comparisons between programs. The cost and burdens of maintenance for a conventional manually curated registry can be great, as exemplified by the United States OPAT Outcomes Registry (OPIVITA) which is no longer operational [2, 9]. In addition, a lack of awareness of the value of OPAT is a common challenge to institutional support of such programs [3]. This is likely due to the fact that most OPAT work is done outside of reimbursable face-to-face patient encounters (eg, reviewing laboratory results, calls to patients and facilities, and addressing complications).

Our own institution has had a formal OPAT program for several years, and like many others we have struggled to produce both analytic data on our performance and objective data on the value of our OPAT team’s work. Given these limitations, we saw this as an opportunity to build a novel OPAT registry that does not require administrative support (ie, can collect data without need for any manual curation), can display analytics and outcome changes over time, and demonstrates the value our OPAT team brings to the institution. We sought to do this in a way that can be replicated by other OPAT programs, to make it easier to compare and aggregate interinstitutional data in the future. The aims of this project were (1) to build an automated OPAT registry in our electronic medical record (EMR) and (2) to validate the registry against formal record review.

## METHODS

### Human Subjects Research

This project was granted an exemption of annual institutional review board review and a waiver of Health Insurance Portability and Accountability Act (HIPAA) authorization for access to and use of protected health information by the Yale Institutional Review Board. This study did not include factors necessitating patient consent

### Identifying the Registry Population with an “Episode of Care”

The Yale OPAT Program monitors patients discharged from Yale–New Haven Hospital (a tertiary care academic hospital in New Haven, Connecticut) on intravenous antibiotics of any duration and/or long-term oral antibiotics. Patients receiving only oral antibiotics can be enrolled in the program if there is a need for regular laboratory monitoring during therapy (eg, more frequently than every 2 weeks). Our hospital system uses the Epic [10] EMR. We identified the registry population based on the presence of an “OPAT episode of care.” An “episode of care” is an Epic tool that enables a group of encounters with a patient (virtual, by phone, or in person) to be associated with a single defining condition. In this case, the activation of an OPAT episode of care allows the patient, hospital encounter, and subsequent outpatient encounters to be associated with outpatient antibiotic therapy. A flowsheet form (a pretemplated form in Epic) linked to this episode of care was created to aide in the transitions of care by standardizing documentation and autopopulating discharge paperwork with recommendations. The selections entered in the flowsheet are stored as discrete data in the EMR, allowing for tracking.

**Figure 1.**
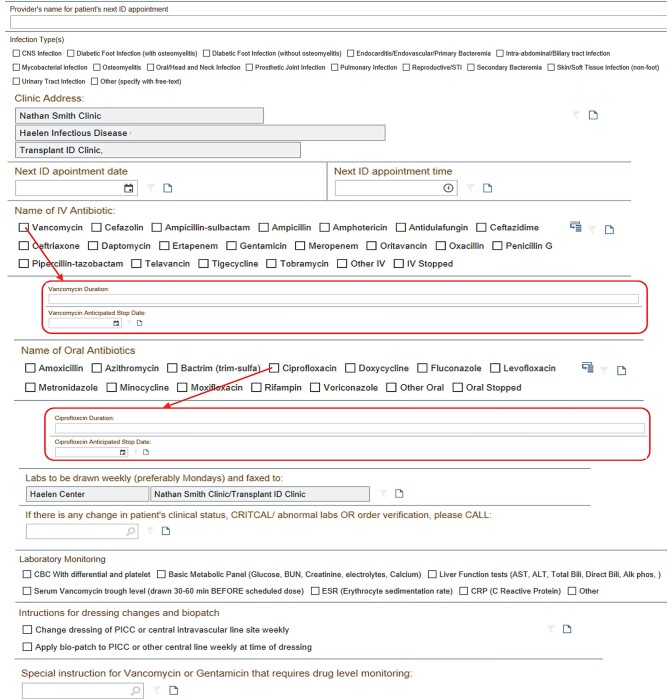
Outpatient parenteral antimicrobial therapy (OPAT) episode flowsheet form. Abbreviations: Alk phos, alkaline phosphate; ALT, alanine aminotransferase; AST, aspartate aminotransferase; Bili, bilirubin; BUN, blood urea nitrogen; CBC, complete blood cell (count); CNS, central nervous system; ID, infectious diseases; IV, intravenous; PICC, peripherally inserted central catheter; STI, sexually transmitted infection; trim-sulfa, trimethoprim-sulfamethoxazole.

The episode of care and a flowsheet form are integrated into our OPAT workflow as follows. First, the ID consultant activates an OPAT episode of care when finalizing recommendations for a patient who will require antibiotic monitoring after discharge from the hospital. Next, the ID consultant uses a flowsheet form (Figure 1) to record the infection types being treated, clinic contact information, names and durations of antibiotics, laboratory monitoring recommendations, and care instructions for the vascular line. Finally, once the OPAT monitoring period is completed, a member of the OPAT team “resolves” (deactivates) the episode.

For the current study we included any patient in our hospital's EMR from 1 January 2019 through 6 January 2023 with an OPAT episode of care whose status was “resolved.” Episodes were excluded from the registry if they were not started during an inpatient admission, if the episode duration was <2 day, or if the hospital discharge disposition was listed as “expired.” Patients with multiple OPAT episodes had each episode included.

### Variable and Outcome Collection and Data Visualization

We selected variables and outcomes based on those reported elsewhere [2, 11, 12] and their availability as reliable discrete data in the EMR (variable definitions are provided as Supplementary Materials). Demographic and descriptive variables included age, sex, race, ethnicity, primary insurance payor, antibiotic names, infection syndromes treated, discharge disposition, dates of hospital admission, discharge, and death. Payors were categorized into 4 groups: Medicare, Connecticut Medicaid, Department of Veterans Affairs (VA), and other. Outcome measures included the 30-day all-cause readmission rate, the 30-day all-cause death rate, complications during OPAT, and an estimate of the hospital days saved by the presence of our OPAT program. The number of hospital days saved was estimated by taking the sum of the durations of the intravenous antibiotic episodes, a method used by others [1, 2, 6, 13]. Episodes with only oral antibiotics were excluded from this calculation as they would not require ongoing hospitalization for antibiotics alone.

Complications during OPAT are identified using a provider-reporting system created in Epic at Yale in 2017. When an OPAT-associated complication occurs, a provider (typically a member of the OPAT team) documents a note in Epic containing pretemplated SmartLists (structured selectable lists with trackable data elements). The lists of complications to select from include catheter-associated complications (catheter thrombus, central catheter–associated bloodstream infection), laboratory abnormalities (missed or incorrect dose of antibiotics, abnormal vancomycin level, acute kidney injury, abnormal complete blood cell count, and abnormal liver function test results or hepatitis), and other adverse drug events (including rash, wrong antibiotic administered, missed laboratory result, *Clostridioides difficile* infection, and diarrhea).

These variables and outcomes were mined using structured query language (SQL) code to extract the data from the Epic Clarity database (a relational reporting database updated daily with data from the Epic operational environment) into a custom table. SQL code was also written to pull the data from these custom tables into a spreadsheet-style tool in Epic (known as Reporting Workbench) to perform the summary analyses. Finally, we created a Radar Dashboard (Epic data visualization tool) to display all the Reporting Workbench analyses in a single location. Data for the registry refresh automatically on a weekly basis.

### Data Validation

A random selection of patients and their associated OPAT episodes who met the criteria for the registry were chosen for the validation. The random sample was supplemented to ensure that some patients met the criteria for death or readmission or had a complication during OPAT so that these variables could be validated appropriately. A convenience sample of 146 episodes (representing 99 patients) was used to validate the registry. When these 146 episodes were reviewed, 9 were excluded (it was discovered during manual record review that, despite having an episode completed, these patients were not discharged on antibiotics that required monitoring). This left 137 episodes in the final validation analysis. All variables and outcomes noted above were validated by comparing the registry output with the relevant clinical notes, forms, and demographic data for each of the 137 episodes. Validation notes are included in the Supplementary Materials.

### Statistical Analysis

Demographic, clinical, and outcomes data were described using sums, means, and standard deviations (SDs). The accuracy of the registry data was assessed in a binary fashion (as correct or incorrect) for each variable of each episode. The frequency of correct data was calculated to determine the accuracy of each variable.

## RESULTS

### Descriptive Data

Over the 4-year time frame, our registry collected 3956 unique patients who completed 4710 episodes (approximately 1200 episodes per year); 584 patients (14.8%) had >1 antibiotic episode. The mean patient age (SD) was 61.1 (15.7) years; 61.7% were male, 68.5% white; and 88.3% non-Hispanic (Table 1). The largest age group was 60–69 years (26.5% of patients) followed by 50–59 years (20.8%) and 70–79 years (20%). The primary payor distribution for the 4710 episodes was as follows: Medicare, 1444 patients (30.6%); Connecticut Medicaid, 1107 (23.5%); VA, 48 (1%); and other, 2094 (44.4%) (unknown in 17 [0.5%]). The mean episode duration (SD) was 26.6 (21.2) days. The most frequent duration was 4–6 weeks (29.8%), followed by 2–4 weeks (24.6%). Intravenous antibiotics were prescribed in 4315 episodes (91.6%), and oral antibiotics in 1584 (33.6%). The most common infection syndrome was osteomyelitis (Table 2; 24.7% of episodes with a reported syndrome). The most common intravenous antibiotic prescribed at discharge was vancomycin (Table 3; 31.8% of episodes). The most common discharge disposition was to home (55.5% of episodes), followed by skilled nursing facility (34.3%) and “other” (10.2%). An image of the dashboard as it appears in Epic (displaying all of the registry data) is provided in the Supplementary Materials.

**Table 1.** Patient Demographic Information

Characteristic	Patients, No. (%)^a^ (n = 3956)
Age, mean (SD), y	61.1 (15.7)
Sex^b^	
Male	2440 (61.7)
Female	1515 (38.3)
Race	
White	2710 (68.5)
Black or African American	677 (17.1)
Other^c^	569 (14.4)
Ethnicity	
Non-Hispanic	3493 (88.3)
Hispanic	423 (10.7)
Other^d^	40 (1)

Abbreviation: SD, standard deviation.

^a^Data represent no. (%) of patients unless otherwise specified.

^b^Sex was listed as unknown for 1 patient.

^c^Including patients who reported >1 race.

^d^Including patients responding “prefer not to share” or “I do not know.”

**Table 2.** Infection Syndromes Treated

Infection Syndrome	Episodes, No. (%) (n = 4015)^a^
Osteomyelitis	990 (24.7)
Endocarditis, endovascular, or primary bacteremia	802 (20.0)
Other^b^	592 (14.7)
Secondary bacteremia	550 (13.7)
Diabetic foot infection (with osteomyelitis)	437 (10.9)
Prosthetic joint infection	302 (7.5)
Intra-abdominal or biliary tract infection	283 (7.0)
Skin and soft-tissue infection (nonfoot)	219 (5.5)
CNS infection	186 (4.6)
Urinary tract infection	124 (3.1)
Pulmonary infection	95 (2.4)
Oral/head and neck infection	69 (1.7)
Diabetic foot infection (without osteomyelitis)	31 (0.8)
Mycobacterial infection	4 (0.1)
Reproductive/sexually transmitted infection	3 (0.1)

^a^Percentages represent the proportion of episodes; because >1 infection syndrome can be selected for a single episode, these percentages do not add up to 100%. The total (denominator) is the total number of episodes in which an infection syndrome was identified.

^b^Syndromes in the “other” category include invasive sinusitis with cerebral abscess, infected seroma, ocular and neurosyphilis, nondiabetic methicillin-resistant *Staphylococcus aureus* and *Staphylococcus lugdunensis* infection, and Right Lower Extremity graft infection.

**Table 3.** Antibiotics Prescribed at Discharge

Antibiotic	Episodes, No. (%) (n = 4710)^a^
Intravenous	
Vancomycin	1498 (31.8)
Ceftriaxone	877 (18.6)
Cefazolin	719 (15.3)
Oxacillin	279 (5.9)
Piperacillin-tazobactam	268 (5.7)
Other intravenous^b^	225 (4.8)
Ertapenem	222 (4.7)
Daptomycin	212 (4.5)
Ceftazidime	194 (4.1)
Ampicillin-sulbactam	156 (3.3)
Penicillin G	136 (2.9)
Ampicillin	124 (2.6)
Meropenem	55 (1.2)
Tigecycline	52 (1.1)
Anidulafungin	46 (1.0)
Gentamicin	37 (0.8)
Amphotericin	20 (0.4)
Oral	
Other oral^c^	623 (13.2)
Metronidazole	590 (12.5)
Ciprofloxacin	522 (11.1)
Rifampin	138 (2.9)
Moxifloxacin	56 (1.2)
Levofloxacin	27 (0.6)

^a^Percentages represent the proportion of episodes. Because >1 antibiotic can be selected for a single episode, these percentages do not add up to 100%.

^b^Intravenous antibiotics listed in the “other” category included telavancin, tobramycin, and oritavancin.

^c^Oral antibiotics listed in the “other” category include voriconazole, isavuconazole, minocycline, fluconazole, azithromycin, linezolid, trimethoprim-sulfamethoxazole, and amoxicillin.

### Outcomes

The 30-day all-cause readmission by year of episode completion was stable from 2019 through 2021, at approximately 21%, and then declined to 16.7% in 2022. The 30-day all-cause death rate by year of episode completion remained stable at approximately 2%. A total of 400 complications during OPAT were documented (Table 4). The most common was “missed/incorrect dose of antibiotics” (32.0%). The registry calculated that the Yale OPAT program was able to reduce hospital length of stay by 114 606 days over 4 years.

**Table 4.** Complications During Outpatient Parenteral Antimicrobial Therapy

Complication	Complications, No. (%) (n = 400)
Missed/incorrect dose of antibiotics	128 (32.0)
Other^a^	88 (22.0)
Abnormal vancomycin level^b^	49 (12.3)
Acute kidney injury	46 (11.5)
Rash	22 (5.5)
Abnormal complete blood cell count	20 (5.0)
Abnormal liver function test results/hepatitis	20 (5.0)
Catheter thrombus	10 (2.5)
Wrong antibiotic	8 (2.0)
Missed laboratory result	6 (1.5)
*Clostridioides difficile* infection	3 (0.8)
Diarrhea	0 (0)
Central catheter–associated blood stream infection	0 (0)

^a^Complications in the “other” category include elevated creatinine kinase level, back pain, elevated creatinine level, hyperkalemia, and itching. On review of the complications reported in this category, 93 actual complications were identified (some of the 88 included >1 documented complication), and 19 of the 93 (20%) could have been listed in a more specific category (eg, elevated creatinine level could have been categorized as acute kidney injury).

^b^Abnormal vancomycin level is defined as a trough level <10 or >15 ug/mL.

### Data Validation

Of the 146 antibiotic episodes, 137 were included in the final validation analysis, giving the registry an accuracy of 93.8% for identifying patients discharged for monitoring by the OPAT team. All variables had an accuracy of >90% (see Table 5). Manual record review revealed that the number of hospital days saved was actually 22.5% shorter than calculated by the registry. Therefore, the 114 606-day reduction in hospitalization over 4 years becomes 88 820 days after applying this correction, or roughly 22 000 days per year.

**Table 5.** Data Validation

Variable^a^	Accuracy, % of Episodes (No. Validated/Total No) (n = 137)^b^
Age	100 (137/137)
Sex	100 (137/137)
Race and ethnicity	100 (137/137)
Primary payor	99.2 (123/124)
Discharge disposition	94.2 (129/137)
Intravenous antibiotic name	93.4 (128/137)
Oral antibiotic name	97.1 (133/137)
Infection syndrome	91.0 (101/111)
30-d readmission rate	96.4 (132/137)
30-d mortality rate	100 (137/137)
Complication during OPAT SmartList capture^c^	99.3 (136/137)

Abbreviation OPAT, outpatient parenteral antimicrobial therapy.

^a^See the Supplementary Materials for additional notes on how data validation was performed for each variable.

^b^The variables primary payor and infection syndrome had different totals. See the Supplementary Materials for details.

^c^This accuracy value reflects whether the registry was correct in identifying whether a complication SmartList was used.

## DISCUSSION

This work shows that it is possible to build a highly accurate automated OPAT registry to collect and display important descriptive and outcomes data for an OPAT program. It also provides evidence for the value of the OPAT team to our patients and institution. By accomplishing this, we address 2 significant concerns many ID providers have about the current practice of OPAT: availability of local data analytics and raising awareness of the value of OPAT [3].

When comparing our registry's output to more than a dozen articles published on OPAT registry data [1, 2, 4–8, 13–23], we found that it makes significant contributions to the existing literature. It turned out to be one of the most comprehensive in the surveyed literature. This is likely a result of its automated nature, allowing rapid collection of a large breadth of data without administrative work. With nearly 5000 antibiotic episodes, our population represents one of the larger OPAT cohorts published, with only 3 publications having a larger cohort [2, 5, 20]. One-third of the registries described the racial demographics of their population [5, 14, 17–19, 21], with ours having one of the largest groups of nonwhite patients (approximately 1200 patients). Only 2 publications reported on payor information [4, 17], making our registry the largest population of Medicaid patients reported on (approximately 1100 OPAT episodes). Of the studies surveyed, ours was also the largest group of patients discharged to a skilled nursing facility (approximately 1600 OPAT episodes). Patients discharged to home and those discharged to skilled nursing facilities represent very different patient populations, so the ability to obtain and distinguish between data gathered from each is an advantage for our registry.

Our estimate of hospital days saved (approximately 88 000) is significantly more than that of other publications [1, 6, 8]. Given the monetary cost of a single hospital day, this has important implications for the healthcare system, as well as for patient satisfaction. We acknowledge that this calculation does not account for the fact that some patients treated with intravenous antibiotics would be switched to oral if the only option was to remain in the hospital for the duration of their treatment. While the ability to discharge patients on intravenous antibiotics to nursing facilities contributes to the reduction in length of stay, we elected to include them in the calculation because our OPAT team is responsible for managing their therapy at these facilities. In addition, we report on the frequency of oral antibiotics, which is uncommon in the literature [19, 20, 22].

Compared with an average of 15%, our 30-day all-cause readmission rate was approximately 21% for the first 3 years of data collection and then fell to 16.7% by the final year. This coincides with the receding coronavirus disease 2019 pandemic, but the prepandemic readmission rate was still higher than in 2022, so this doesn’t explain the findings. There is a correlation with staffing improvements made to the OPAT team (more nursing and pharmacy support) by the end of this period, which suggests that more OPAT staff may improve the safety of our patients. This remains only an observation, however, until we can distinguish causes of readmission (OPAT related vs non–OPAT related) and control for severity of illness.

To date, we have presented the registry data at our institution's OPAT, ID section, and administrative meetings. Our goal is to use these data to drive quality improvement work. For example, the identification of the high rate of vancomycin use in our program is an important observation because it has been shown to cause more adverse events than patients receiving other antibiotics (eg, daptomycin) [24]. Quality improvement initiatives have been undertaken to reduce vancomycin usage and the registry is being used to monitor this. By building a registry on a platform that can be replicated by other OPAT programs, we hope this facilitates aggregation and comparison of data across institutions (something that has been lacking to date) and addresses the absence of interinstitutional standardization of OPAT data and outcomes. We have begun working with OPAT programs across the country to strategize how these tools can be replicated at their institutions. Just as Alan Tice, one of the pioneers of OPAT, wrote more than 20 years ago, “Every field of medicine is looking for outcome measures that are useful in assessing the quality of care and justifying the therapies provided” [25]. Aggregated data from multiple OPAT registries could be used to gain insights into the risks of readmission [26, 27] and complications as well as to suggest new treatment strategies [2].

The strengths of this registry include the size and diversity of data (patients of color, Medicaid recipients, and facility-based patients). Having more data on these OPAT groups is important. As Billmeyer et al showed [19], patients receiving OPAT who identify as black or African American had an increased odds for severe adverse drug reactions. The in-depth validation of the data are also a strength. Our method of tracking complications during OPAT has both strengths and limitations. The fact that it relies on documentation via an Epic SmartPhrase rather than mining laboratory and coding data carries the advantage of being able to track complications that occur outside our health system. It is, however, limited by human error (eg, forgetting to document a complication or documenting under the wrong category). For example, some complications documented in the “other” category could have been listed in a more specific group (eg, acute kidney injury). On review, this was true only 20% of the time, suggesting that individuals were generally accurate with their categorizations. OPAT complication rates are widely variable in the literature, likely owing to lack of standardization as to what constitutes an OPAT complication. Some studies have rates as low as 4.6% [2], while others are as high as 52% [19].

Our complication rate was on the lower end of this spectrum (averaging approximately 1 complication for every 12 episodes), but we suspect that this may be due to reporting bias. For example, no central catheter–associated bloodstream infections were identified with this system, even though they certainly occurred. This may have been because these patients required readmission, bypassing the adverse event reporting mechanism if the OPAT team was not made aware. To help this situation, in the future the complication reporting system may be supplemented with discrete data measures (eg, *C difficile*–positive stool test results).

Another limitation is that our registry does not collect outcomes on infection cure versus relapse versus treatment failure. Our priority was to provide automized data collection and given the inability to reliably extract these outcome data without manual record review, they was excluded from this version of the registry. Moreover, the registry currently lacks measures of severity of illness (eg, the Charlson comorbidity index), thereby limiting our ability to interpret observations in outcomes. We intend to include these in future updates. Our registry requires the Epic EMR system, specifically the OPAT Episode of Care and flowsheet form information. This potentially reduces its exportability to other institutions. However, the features that we have established here can be used to help design similar registries in Epic and other EMR systems and provide an avenue for future work. Finally, the exclusion of patients whose OPAT was started in the ambulatory setting likely had little impact on the results, as they represent approximately 8% of total episodes per year.

In the months since this registry has been available, we have used our experience to make adjustments: including additional measures of OPAT teamwork volume, improving the granularity of data captured on antibiotic names and complications, and improving our ability to associate complications with specific antibiotics and skilled nursing facilities. These improvements will serve as the foundations of future research into the value of OPAT and drive local quality improvement. We intend to work with Epic to make this registry build available to others. In the meantime, OPAT programs interested in reproducing this registry should consider having access an EMR analyst trained in SQL programming to help adapt our methods of automation.

## Supplementary Material

ofae004_Supplementary_Data

## Supplementary Data

Supplementary materials are available at *Open Forum Infectious Diseases* online. Consisting of data provided by the authors to benefit the reader, the posted materials are not copyedited and are the sole responsibility of the authors, so questions or comments should be addressed to the corresponding author.
