# Supplementary material for: Creation and Validation of an Automated Registry for Outpatient Parenteral Antibiotics
Source: Open Forum Infect Dis. 2024 Jan 12;11(2):ofae004. doi: 10.1093/ofid/ofae004 (PMC10866572; doi:10.1093/ofid/ofae004)
Supplement: ofae004_Supplementary_Data [file ofae004_supplementary_data.docx]

**Supplemental Materials**

**
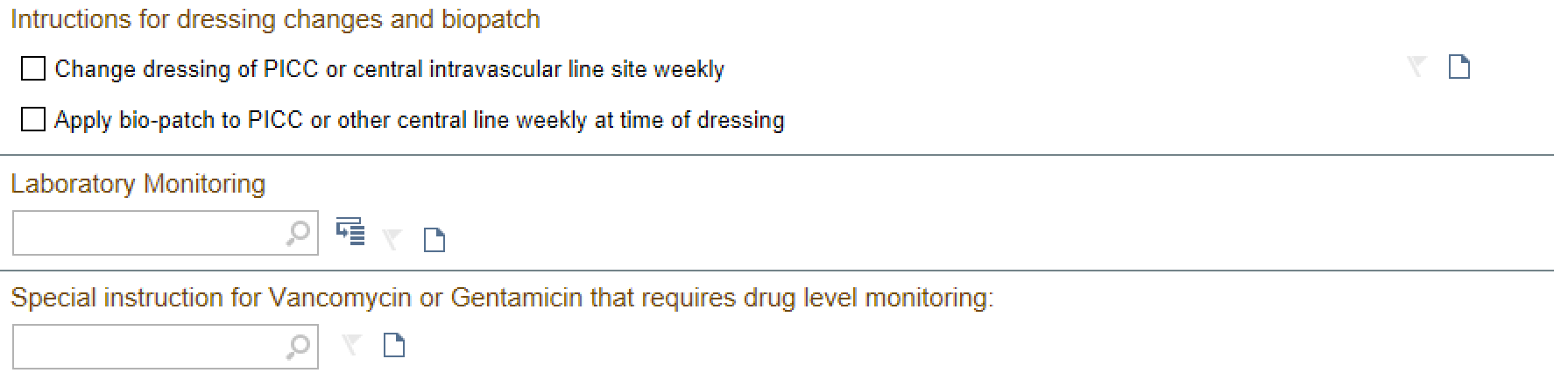

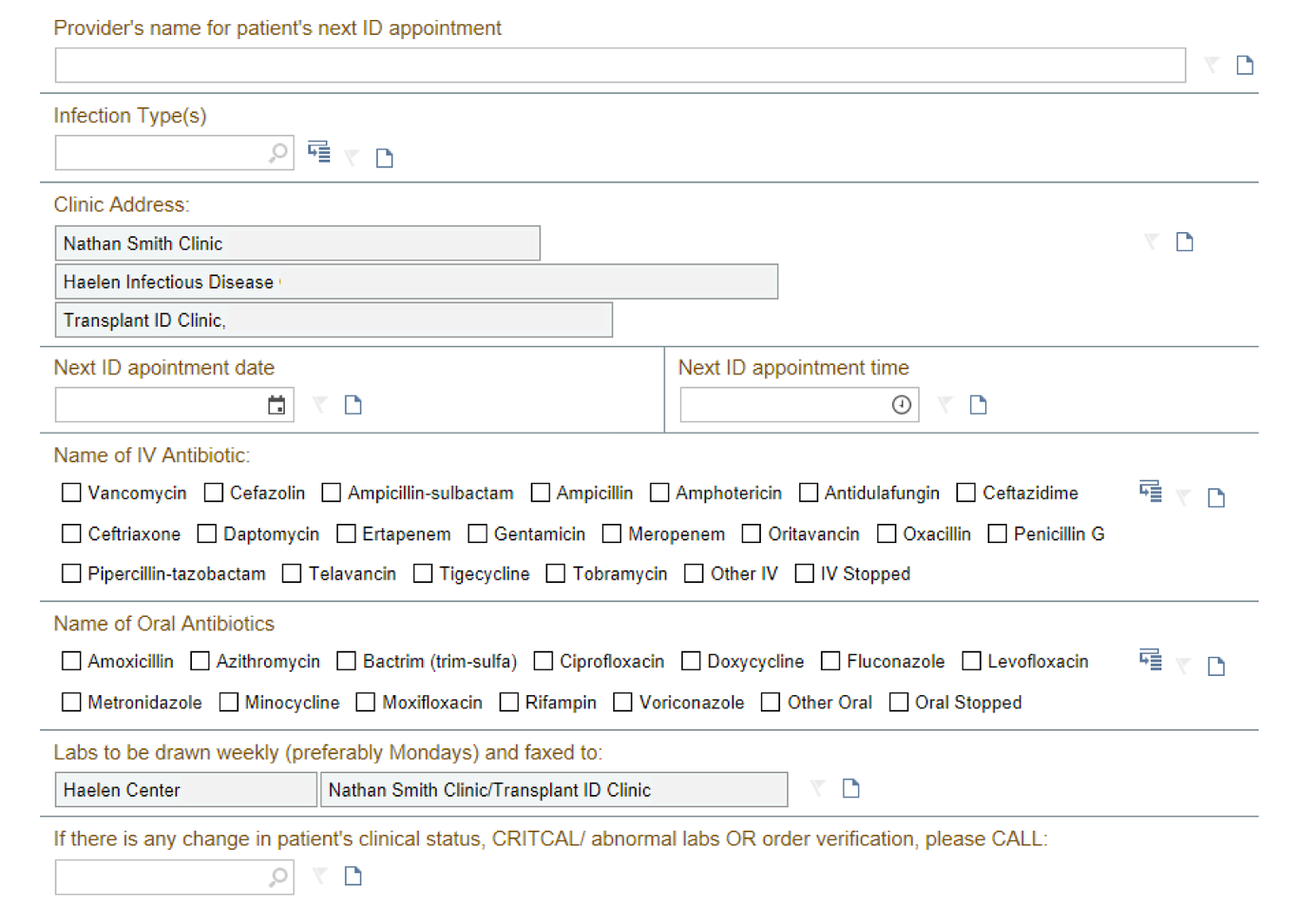
S1: OPAT Episode Flowsheet Form**

**S2: Variable Definitions**

| Variable | Definition |
| --- | --- |
| **Total Number of Unique Episodes** | The number of unique episodes that meet the inclusion/exclusion criteria above. |
| **Total Number of Unique Patients** | The number of unique patients that have episodes that meet the inclusion/exclusion criteria above. |
| **Number of Patients with More than One Episode** | The number of “unique patients” (from the above variable) that have more than one OPAT episode. |
| **Episode Duration** | The number of days between the “anchoring hospitalization” discharge date (the hospitalization which the OPAT episode was linked to) and the episode end date (defined as the date the episode status was switched to “resolved”, re-admission to the hospital, or death (whichever came first)). |
| **Age** | The age of the patient (in years) on the date of discharge from the anchoring hospitalization discharge of each episode. |
| **Sex** | Group the listed sex as “Male” or “Female”. Any other entry was grouped under “Other”. |
| **Race** | Group the listed race as “White” or “Black or African American”. Any other entry was grouped under “Other”. |
| **Ethnicity** | Group the listed ethnicity as “Hispanic” or “Non-Hispanic”. Any other entry was grouped under “Other”. |
| **Infection Syndromes Treated** | Retrieved from the infection syndrome discrete data selection(s) in the episode of care |
| **IV Antibiotics Used** | Retrieved from the IV antibiotic discrete data selection(s) in the episode of care. |
| **Oral Antibiotics Used** | Retrieved from the oral antibiotic discrete data selection(s) in the episode of care. |
| **Hospital Discharge Disposition** | The discharge disposition as listed in the discharge summary of the anchoring discharge. |
| **Payor Source** | The primary payor of the hospital account linked to the triggering admission of the OPAT episode. |
| **Estimated Hospital Days Saved** | The sum of the episode durations for those patients who had one or more IV antibiotics prescribed at discharge (patients discharged on oral antibiotics alone would not require ongoing hospitalization for this alone). |
| **30-Day Re-admission Rate** | Count as “yes” if the patient had a re-admission to the hospital ≤ 30 days from the anchoring hospital discharge date based on CMS unplanned 30-day readmission criteria (including observation stays). |
| **30-Day Death Rate** | Count as “yes” if the patient died ≤ 30 days after the anchoring hospital discharge date. |
| **Adverse Events** | The number of times the OPAT adverse event Smart List was used within an episode duration start and end timeframe. Display the discrete data selection chosen from the Smart List. |

**S3: Data Validation Notes**

| **Dashboard Report** | **Notes** |
| --- | --- |
|  |  |
| **Age** |  |
| **Sex** |  |
| **Race & Ethnicity** |  |
| **Primary Payor** | Able to confirm payor for 124 of 137 episodes. For the one incorrect value, the report pulled the secondary insurance instead of the primary insurance. |
| **Discharge Disposition** | Disposition not listed in report (n=1), disposition incorrect on report (n=2), abbreviation used for disposition resulted in episode being placed in the “other” category instead of “SNF” category (n=5). |
| **IV Antibiotic name** | Patient was discharged on oral antibiotics only, but report lists IV (n=5), two IV listed on report but only one is correct (n=2), incorrect IV name (n=2) |
| **Oral Antibiotic name** | Patient was not discharged on oral antibiotics, but report lists an oral antibiotic (n=3), patient was discharged on an oral antibiotic that was not listed in the report (n=1) |
| **Infection Syndrome** | 111 of 137 episodes with an infection syndrome provided. 10 episodes were mis-classified: patients with diabetic foot infection with osteomyelitis that were classified as “osteomyelitis” (n=7) or primary bacteremia/endovascular infections that were classified as “secondary “bacteremia” (n=3). 28 additional diagnoses should have been included but were not documented by the ID clinician in the episode of care. |
| **30-day re-admission** | The 5 patients labeled incorrectly had 30-day readmissions found on chart audit that were excluded from the dashboard report because they did not meet the CMS criteria for 30-day hospital wide readmission. |
| **30-day mortality** |  |
| **Adverse Event smart phrase** | Accuracy calculated based on whether the report was correct in identifying which episodes had or did not have an adverse event smart phrase associated with them. The one incorrect value was the result of the smart list selection being erased and free-texted in (rather than using the discrete data element). |
| **Hospital Days Saved** | Actual=2338 days, dash report=3016 days |
